# Being human in non-human systems: why nursing leadership needs Artful Intelligence™ alongside artificial intelligence

**DOI:** 10.1177/17449871261481731

**Published:** 2026-10-17

**Authors:** Marion Lynch

**Affiliations:** Nurse, Artist, Founder and CEO of Art of Nursing CIC, University of West London, London, UK

## Introduction: The nurse cape

If ever there was a profession called into existence by the real needs of humanity, it is nursing. (Parsons, 1917)

More than a century later, nursing continues to wrestle with visibility, recognition and value. Much of what matters in nursing remains difficult to see, and artificial intelligence (AI) in nursing is both a help and a hinderance to our future. AI is described as a new frontier in nursing (Tranka, 2025), and a way of transforming and strengthening nursing (Yakusheva et al., 2025). Although replacing some tasks, AI will not be able to replace nurses (Ball Dunlap et al., 2026). Or will it? AI is expanding our capacity to process information, predict outcomes and automate tasks in increasing complex inequitable health systems. The questions for nursing leadership are how AI will influence practice, and how will we ensure we remain human in non-human systems?

I use art to explore this further through the Cape of Home, Health and Hope (Figure 1). The cape is an immersive art installation. It is a cape telling a story of nursing. It traces 100 years of nursing across four generations of women in my family (Figure 2). It is stitched with photographs, badges, places and words, red embroidery on white linen, a story of women’s migration and leadership.

**Figure 1.**
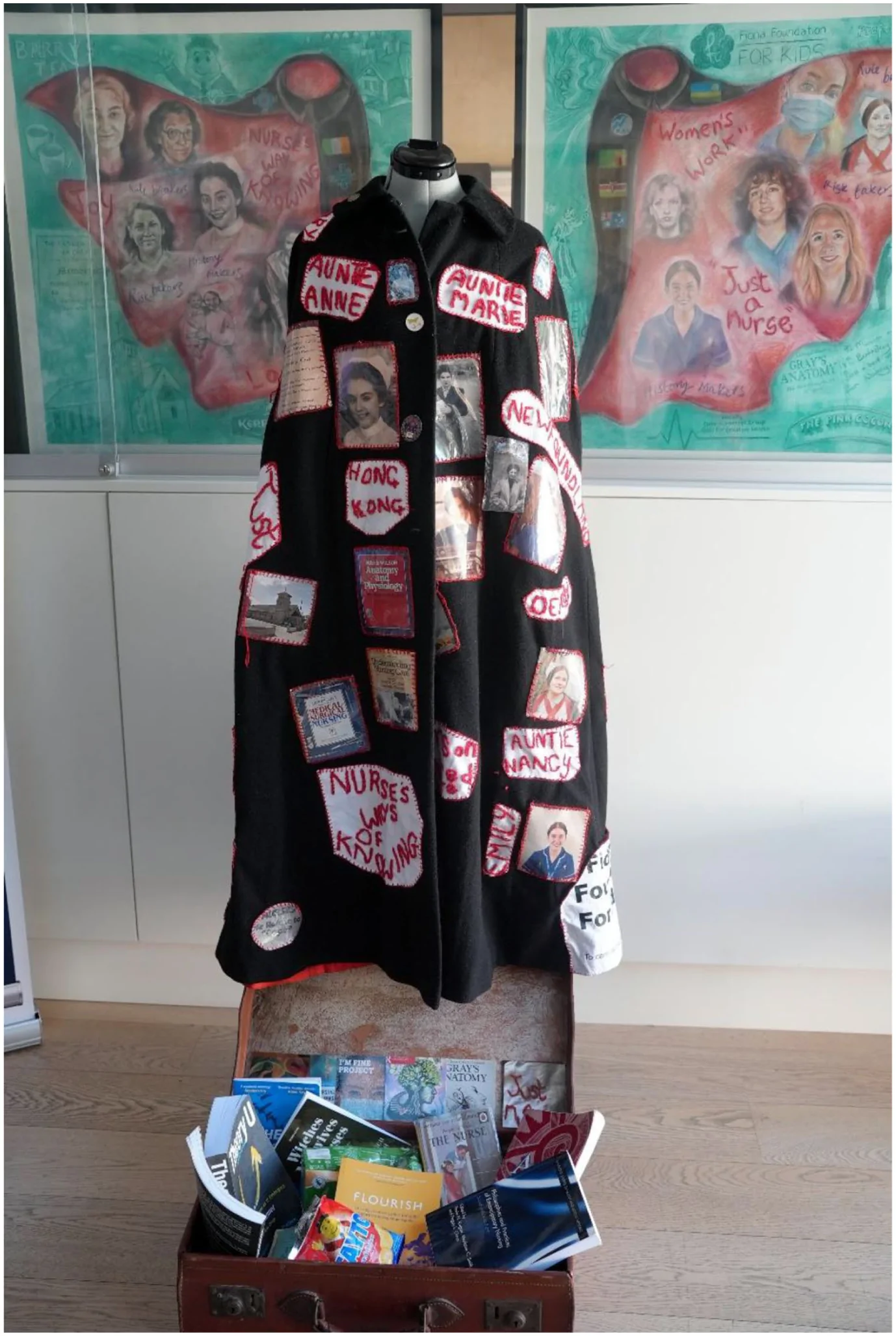
Image of the art installation of Cape of Home, Health and Hope and accompanying artwork showing key places and faces within the story of the cape. Exhibited at the Art of Nursing Exhibition: Nurses: Being seen, being heard, being. May 2026. Clore Learning Centre, Kensington Palace. Artist for the cape Dr Marion Lynch Artist for the pictures Kitty Cameron Credit Erika Harlos-Szendrey

**Figure 2.**
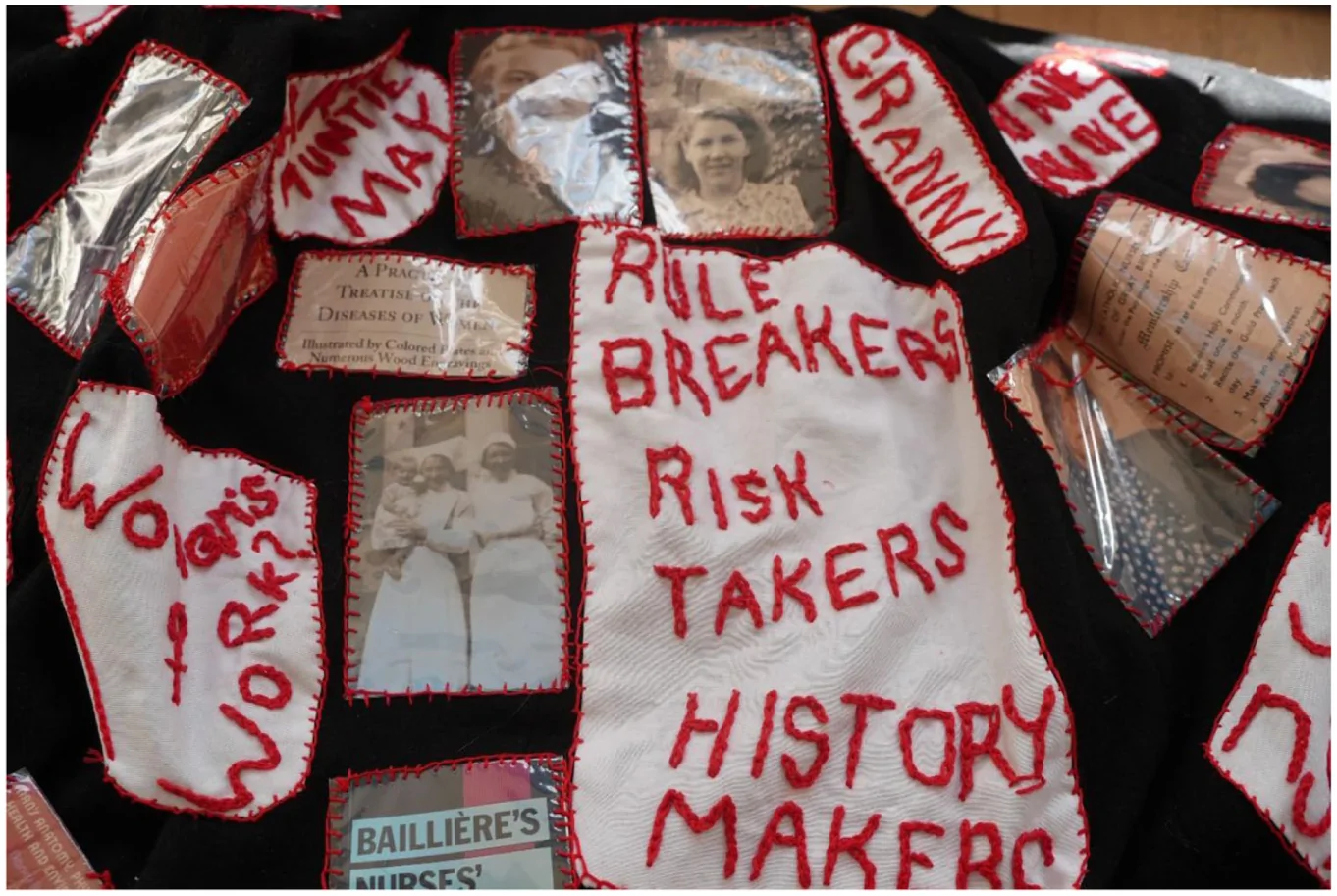
Image from the art installation Cape of Home, Health and Hope. The words spoken by nurses, family photographs of nurses and images of books from nurse training across a century, all interwoven and stitched onto the cape. Artist Dr Marion Lynch Credit Erika Harlos-Szendrey

Nurses want to wear it. When they wear it, they speak of the weight. Then, they share memories, they had a cape. Wishes, they wanted a cape. And worries. They share what it means to be a nurse. The cape is a boundary object, an artefact creating dialogue across experiences and generating knowledge that cannot emerge through words alone (Daykin, 2020).

The cape conversations move to career conversations. Embodying nursing through these lived experiences highlights how nursing leadership is in relationships not organisational charts. It builds through shared values, collective stories and a commitment to nursing and human flourishing. The cape experience makes visible the many significant aspects of nursing difficult to measure, easy to feel and impossible to ignore.

One nurse remembered three hours of sitting silently with a woman whose world was fragmented through severe mental illness. The nurse was the person’s anchor to reality. Had the woman been compulsorily detained, the care would have been counted. The nursing presence that prevented further deterioration was not. The cape made it possible to say it.

Such moments expose our nursing leadership challenge. Health systems count nursing activity more easily than relationships.

As we adopt non-human approaches, how will we ensure that the human aspects remain visible, valued and developed? What we notice depends on the lens through which we look.

The biomedical lens reveals disease, treatment and outcomes, professional lens competence and standards, digital lens efficiency and time optimisation. Each offers valuable insight and leaves something unseen. I suggest a kaleidoscope lens allowing us to see more.

Barbara Carper’s (1978) patterns of knowing identified empirical, ethical, personal and aesthetic dimensions of practice, a wider lens on nursing than most see. Almost 50 years later, the empirical science lens is still the dominant discourse on what nurses do.

As a mental health nurse in the 1980s, I knew nursing was more than this and much of what I did could not be codified. Trust and presence building, human connection and meaning-making for the person who needed help to make sense of their situation.

Martin Buber’s concept of the I–Thou relationship provided language for this distinction. Buber (1970) described relationships characterised by genuine encounter rather than objectification. Nursing at its best has always been rooted in this way of being, but this being aspect of nursing is difficult to break down into task or time or be automated by AI. I propose we need a parallel intelligence to AI.

Artful Intelligence™ is the integrated capacity to perceive human experience, exercise ethical judgement, cultivate trusting relationships, interpret narratives and apply imagination in situations of uncertainty. It compliments AI, enabling us to do what AI cannot do.

At its core, Artful Intelligence™ consists of five interconnected dimensions:

- Aesthetic intelligence, the capacity to notice.
- Ethical intelligence, the capacity to judge.
- Relational intelligence, the capacity to connect as people.
- Narrative intelligence, the capacity to understand and make meaning.
- Imaginative intelligence, the capacity to create alternatives for the future.

These are interdependent. Remember the three hours in silence experience. The nurse noticed subtle changes in emotion and behaviour. AI may pick up movement, only the nurse can sense and interpret their significance within the person’s life. She draws upon ethical judgement to determine what is needed and responds sustaining dignity and human connection. This is Artful Intelligence™ in action, this is how presence happens.

So how do nurse leaders balance the human and non-human and ensure both evolve together and sustain us?

Nursing is emotionally demanding (Feng et al., 2024). We occupy the space between organisational expectations, professional obligations and the human realities faced by nurses and those we nurse. We witness suffering, uncertainty and loss while simultaneously managing performance, resources and change. Sustaining ourselves and our leadership roles requires us to manage both the human and the non-human aspects of nursing care.

Contemporary leadership discourse frequently emphasises resilience, yet resilience has become an individualised solution to systemic problems (Webb, 2025). Artful Intelligence™ acknowledges that sustaining leadership depends upon maintaining the human in the non-human system by maintaining connection with meaning, values and relationships.

As with Carper’s ways of knowing in nursing (1978), Artful Intelligence™ makes the invisible visible (Figure 3). If we refer back to the cape, picture it as our visibility cape. The aesthetic dimension enables leaders to notice what is happening beneath the key performance indicators and share this. The ethical dimension supports difficult judgements where values collide and conversations about nursing’s cost and value compete. The relational dimension strengthens trust and psychological safety. The narrative dimension makes sense of complexity. The imaginative dimension enables possibilities to be envisioned when existing solutions do not help, aided using the arts (Lynch, 2026).

**Figure 3.**
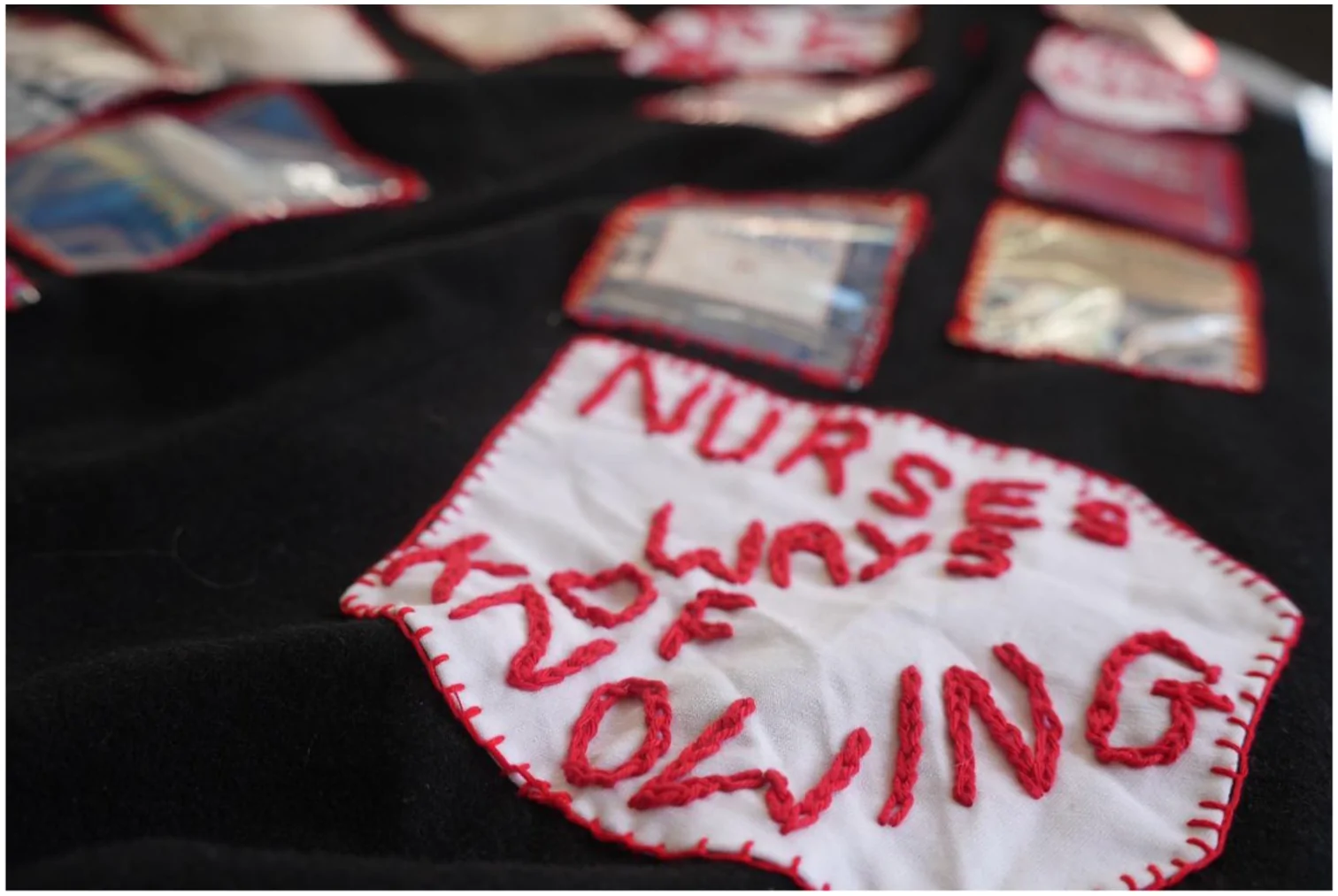
An image from the art installation, Cape of Home, Health and Hope. Carper’s wise words in red embroidery on white linen. Artist Dr Marion Lynch Credit Erika Harlos-Szendrey

This is important because leadership is not just what we do. If we do it well, it is something we embody. When we become disconnected from our own humanity, we struggle to create environments in which others can remain connected to theirs. This is when nursing goes horribly wrong (Francis, 2013). Artful Intelligence™ therefore functions as a professional capability and a source of personal sustainability. It also helps the system thrive.

Complexity science reminds us that outcomes emerge through relationships not linear cause-and-effect processes and that change cannot simply be imposed through plans, policies and targets (Plsek and Greenhalgh, 2001, Uhl-Bien et al., 2020). This complexity lens shifts thinking. Rather than controlling change and people, we need to focus on creating the conditions in which change can emerge and people can flourish.

Scharmer (2016) moved complexity from systems to people and developed Theory U, a framework helping leaders move beyond habitual assumptions and become attentive to emerging possibilities. For nursing this requires a shift in how we think and what we think about ourselves as leaders. U Theory is already informing learning about nursing leadership (Figure 4).

**Figure 4.**
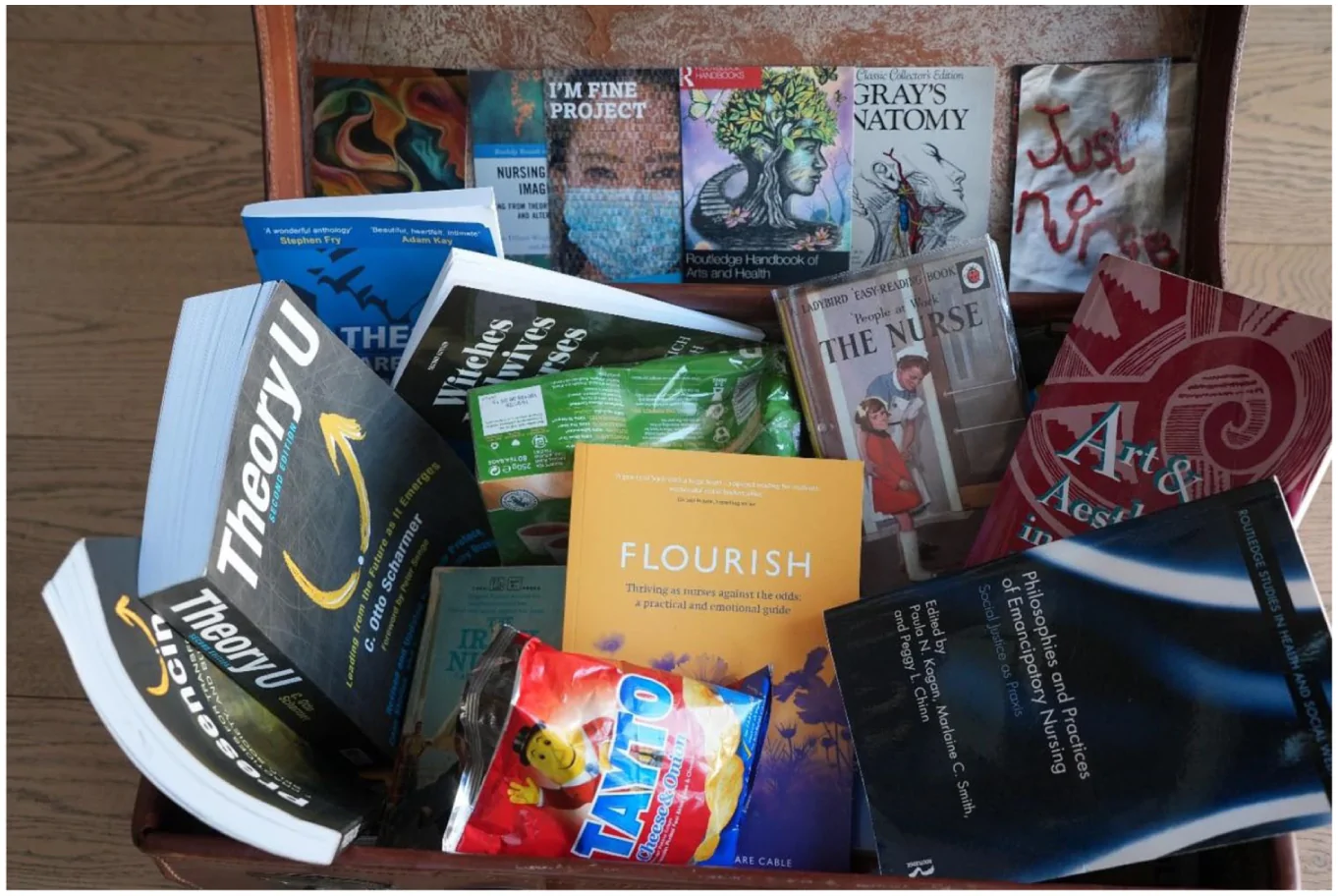
Image from the art installation, Cape of Home, Health and Hope. Image of the suitcase containing books from 1963-2026 and treasured Irish crisps and tea. Artist Dr Marion Lynch Credit Erika Harlos-Szendrey

Like Theory U, person-centred practice requires us to connect with our inner selves as human beings with feelings, emotions, thoughts and desires that guide us as persons (Cable et al., 2024, p. 3167).

The idea is that instead of ‘*downloading*’ solutions from the past, nurse leaders learn to notice what is emerging, stop reproducing existing patterns and focus on cultivating the conditions for innovation, learning and growth.

Artful Intelligence™ is the capability to do this. It enables leaders to engage with complexity without becoming overwhelmed by it and to remain human while leading within non-human systems. It also influences how we influence our environment. This is important because compassionate, person-centred care does not happen because compassionate people work within a system. It requires conditions that support connection, reflection and relational practice (Ryan, 2022). It requires tending.

The role of leadership is therefore ecological as much as operational and the quality of any results depends on the quality of the awareness from which the people in the system operate (Scharmer, 2016). We also require tending and need to attend to cultivating what Scharmer called the ‘social soil’, the invisible, foundational substrate of relationships, trust and shared awareness that underpins all visible outcomes in society and organisations. To do this we need:

1. Awareness: Leadership does not start with a vision; it starts with becoming aware and tuning into other people’s perspectives.
2. Listening: It is so easy to get out of touch as a leader, stuck in our own bubble.
3. Dialogue: This is more than talking; it is creating the capacity of a system to see itself.

We know we cannot change a system unless we change the mindsets of those creating the system. The almost impossible task, and a challenge for all system leaders is to be able to listen without controlling, notice what we are protecting and tolerate not knowing long enough for something new to appear (Ratanjee, 2026). Impossible until it is done.

Artful Intelligence™ is a frame to help us do this and cultivate the social soil of healthcare. Aesthetic intelligence will help us see what is invisible without this new thinking. Narrative intelligence enables our experiences to be interpreted and shared, addressing forms of epistemic injustice that have historically marginalised nursing knowledge. Imaginative intelligence enables futures to be envisioned that do not yet exist and are not built on past thinking. This is a collective not individual endeavour, and as with complexity, needs networks and metaphors to make it make sense.

The cape is an artifact and also a metaphor. The future of nursing will undoubtedly involve AI. Much of what we do will be automated, augmented or redesigned. But some aspects cannot be outsourced to AI. These are the social soil of nursing; theinvisible and vital elements of trust, compassion, belonging, hope and meaning-making.

The Cape of Home, Health and Hope is an art installation (Figure 5), an artifact that enables nurses to imagine how the future will emerge. While wearing this century of nursing current nurses were asked; what words should stay on this cape, what should go and what future ways of knowing are needed?

**Figure 5.**
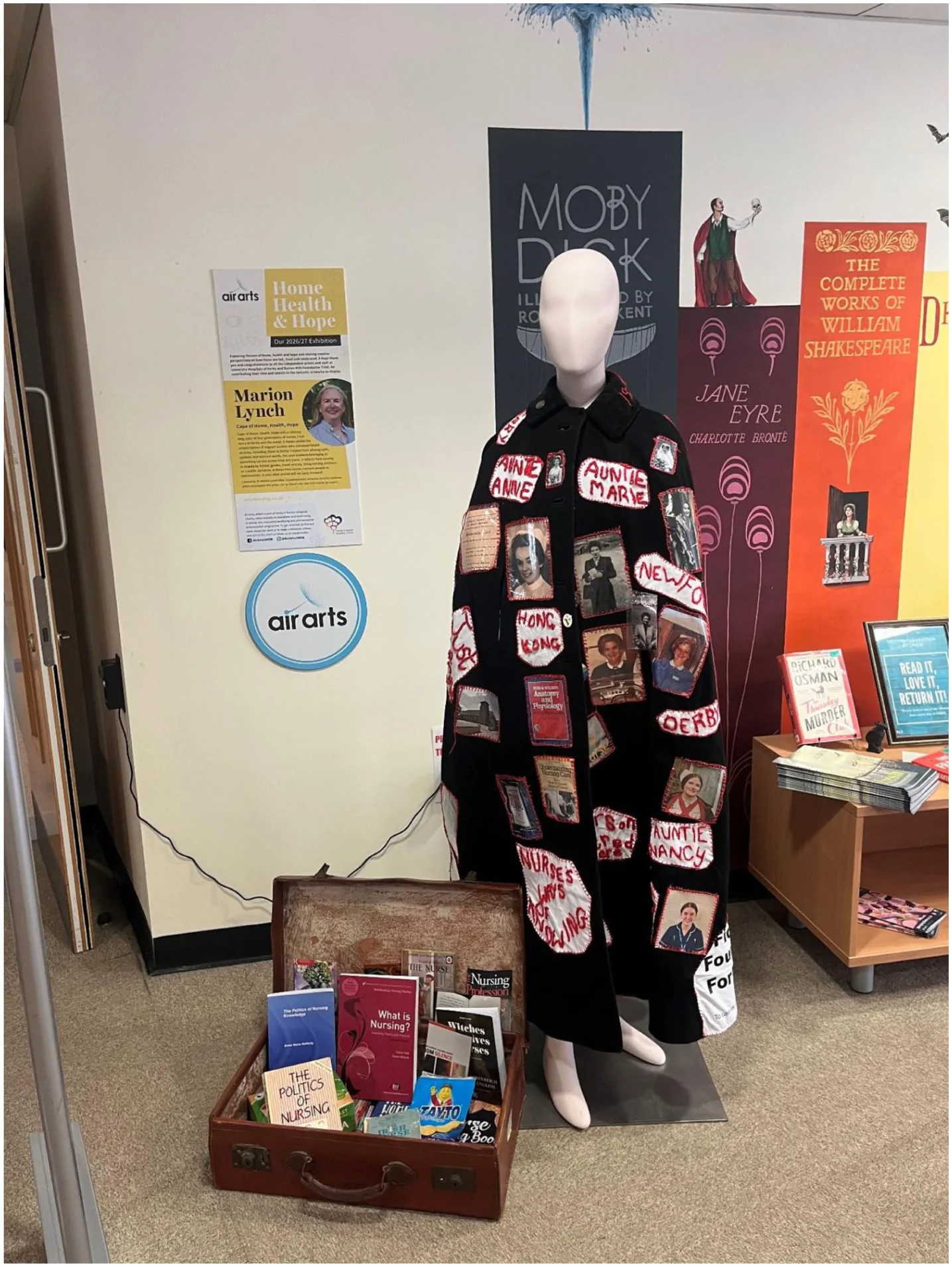
Cape of Home Health and Hope. In situ in Royal Derby Hospital June 2026–2027 as part of the Exhibition. Home, Health and Hope. Artist Dr Marion Lynch Credit Kathryn Stoppard

The future of nursing leadership does not lie in choosing between AI and Artful Intelligence™. It lies in ensuring they develop together. It lies in developing both the art and science of nursing and using the arts and science to imagine 21st century nursing care.

Without all of these, nursing risks losing our human element.

Our leadership challenge is to nourish the ‘social soil’ of nursing, create the conditions in which human, Artful Intelligence™ and AI work together, and keep the human in non-human systems.
